# An Overview of Autism Spectrum Disorder, Heterogeneity and Treatment Options

**DOI:** 10.1007/s12264-017-0100-y

**Published:** 2017-02-17

**Authors:** Anne Masi, Marilena M. DeMayo, Nicholas Glozier, Adam J. Guastella

**Affiliations:** Autism Clinic for Translational Research, Brain and Mind Centre, Central Clinical School, Sydney Medical School, University of Sydney, Sydney, NSW Australia

**Keywords:** Autism Spectrum Disorder, Diagnosis, Heterogeneity, Treatment

## Abstract

Since the documented observations of Kanner in 1943, there has been great debate about the diagnoses, the sub-types, and the diagnostic threshold that relates to what is now known as autism spectrum disorder (ASD). Reflecting this complicated history, there has been continual refinement from DSM-III with ‘Infantile Autism’ to the current DSM-V diagnosis. The disorder is now widely accepted as a complex, pervasive, heterogeneous condition with multiple etiologies, sub-types, and developmental trajectories. Diagnosis remains based on observation of atypical behaviors, with criteria of persistent deficits in social communication and restricted and repetitive patterns of behavior. This review provides a broad overview of the history, prevalence, etiology, clinical presentation, and heterogeneity of ASD. Factors contributing to heterogeneity, including genetic variability, comorbidity, and gender are reviewed. We then explore current evidence-based pharmacological and behavioral treatments for ASD and highlight the complexities of conducting clinical trials that evaluate therapeutic efficacy in ASD populations. Finally, we discuss the potential of a new wave of research examining objective biomarkers to facilitate the evaluation of sub-typing, diagnosis, and treatment response in ASD.

## Introduction

Autism spectrum disorders (ASDs) are complex, pervasive, and multifactorial neurodevelopmental conditions. Observation of aberrant behavior forms the basis of diagnosis, with criteria focused on impairments in social communication and interaction, and restricted, repetitive patterns of behavior, interests, or activities [1]. Heterogeneity of presentation is a hallmark [2–4] with comorbid psychiatric and medical morbidities frequently reported. Commonly identified psychiatric and cognitive comorbidities with ASD include social anxiety disorder, oppositional defiant disorder, attention-deficit/hyperactivity disorder, and intellectual disability [5–7]. Medical conditions frequently reported include immune system abnormalities, gastrointestinal disorder, mitochondrial dysfunction, sleep disorders, and epilepsy [8–10].

The substantial direct and indirect effects of ASDs extend across many different sectors including health, education, social care, housing, employment, welfare benefits, and labor markets, with a high economic burden extending to adulthood and often carried by families [11, 12]. With forecasts of annual direct medical, non-medical, and productivity costs projected to reach close to $US 500 billion by 2025 in the United States alone [13], the importance of adequate care, support structures for affected individuals and their families, and efficacious treatments to improve functioning and outcomes cannot be underestimated.

## Diagnosis, Prevalence, and Etiology

In 1943, Leo Kanner published a report entitled “Autistic disturbances of affective contact”, detailing eleven case studies of children (eight males and three females) aged from 2 years and 4 months to 11 years who had presented to his clinics [14]. Kanner described observations of these children as having an extreme inability to relate to others that appeared to be present throughout infancy. Kanner drew a distinction between this syndrome and that of “childhood schizophrenia” based on the time of onset, as childhood schizophrenia was explained as withdrawal following typical development. Along with this desire for aloneness, Kanner also observed unusual language development, with an aptitude for nouns and learning nursery rhymes, a failure to develop the communicative aspects of speech, a tendency to show echolalia, and a tendency to interpret things literally, along with sensory sensitivities and repetitive behaviors.

In 1944, Hans Asperger published a paper describing what he termed “autistic psychopathy”. This paper described children who primarily had difficulties with non-verbal communication and related social skills. This paper would eventually be considered as important as Kanner’s work in the development of the concept of autism, since the core symptoms were the same as those identified by Kanner but in higher-functioning individuals [15, 16]. As it was published in Germany, in German, during the Second World War, it was not widely read and did not enter the English-speaking medical community until the 1970s [17]. In 1981, Lorna Wing provided a history of the syndrome proposed by Asperger, though she renamed it “Asperger syndrome” to remove the connotations of “psychopathy” [17]. She acknowledges in her introduction the similarities between the criteria proposed by Kanner and Asperger, noting “the argument continues as to whether they are varieties of the same underlying abnormality”. Wing described and refined Asperger’s initial diagnostic criteria, and highlighted the continuum in criteria ranging from the lower functioning “Kanner’s autism” to “Asperger’s syndrome” to typically-developing individuals, who display some of the criteria of Asperger’s syndrome [17].

Infantile autism as an endorsed medical diagnosis first appeared in the Diagnostic and Statistical Manual of Mental Disorders, third edition (DSM-III) [18], and described a subgroup of pervasive developmental disorder (PDD) [19]. The criteria outlined for Infantile Autism required an onset prior to the age of 30 months, a failure of responsiveness to others, gross deficits in language development, and bizarre responses to environmental stimuli, with an absence of schizophrenic symptoms. The criteria were broadened in the DSM-III-R to recognize the pervasive nature of the disorder and that it was not limited to infants, as the criteria for Infantile Autism excluded a subgroup of higher-functioning individuals who displayed the deficits described but did not evidence the symptoms early enough in life to receive the diagnosis. The revision of Infantile Autism to Autistic Disorder in the DSM-III-R recognized the broader spectrum of functioning with 8 out of 16 possible criteria required for diagnosis. The age of onset was specified as either during infancy or early childhood, with a childhood onset specifier (after 36 months). These criteria allowed for the identification of potentially less-impaired individuals to receive a diagnosis [20].

The DSM-IV was released in 1994 with criteria similar to the DSM-III-R for the diagnosis of Autistic Disorder, though the childhood onset specifier was removed as onset before 36 months of age was required. The DSM-IV introduced a formal set of criteria for Asperger’s Syndrome using some of the criteria outlined by Wing [17]. The criteria for Asperger’s Syndrome described a condition with impairments in social interaction, communication, and imagination, similar to that described by the Autistic Disorder criteria, but without the impairments in language or cognition [21].

The move to the DSM-5 was marked by broadening of the definition and reduction in the specificity of autism-related symptoms [1], heralding substantial changes to the diagnostic criteria. Diagnoses of Autistic Disorder, Asperger’s Syndrome and Pervasive Developmental Disorder—Not Otherwise Specified (PDD-NOS), were removed as diagnostic classifications and collapsed into two diagnoses, Autism Spectrum Disorder and Social Communication Disorder. This latest modification reflected growing concerns about the validity of the Asperger’s diagnosis, given evidence that it was frequently interchanged across time with Autistic Disorder [22]. Individuals who would have previously received a diagnosis of Asperger’s Syndrome were generally thought to receive a diagnosis of “Autism Spectrum Disorder without language or cognitive impairment” (DSM-5). The current DSM-5 criteria for Autism Spectrum Disorder are listed in Table 1, with specifiers for current severity summarized in Table 2 [1]. For DSM-5, Social (Pragmatic) Communication Disorder (SCD) was introduced and required persistent difficulties in the social use of verbal and nonverbal communication, without impairments relating to restricted, repetitive behavior. It is expected that those who were previously diagnosed as PDD-NOS, and do not meet the DSM-5 Autism Spectrum Disorder criteria, would be more frequently diagnosed with SCD [23]. The new criteria were expected to enable greater standardization of diagnosis. Previously, a multisite observational study revealed significant variation in clinical diagnoses of specific ASDs, despite similar distributions of scores on standardized measures across sites, supporting the transition from the subgroupings used in DSM-IV to the current dimensional descriptors of the core features of social communication and interaction, and restricted, repetitive behaviors [24]. With the release of DSM-5, it is clear that many of the debates initiated by Kanner’s, Asperger’s, and Lorna Wing’s work remain. There continues to be great debate about the number of different diagnoses with the term Autism Spectrum Disorder, the lack of clarity over the relationship between functioning levels of autism and impaired cognitive function, and the diagnostic relevance and need for treatment for those individuals who appear to show higher levels of occupational and intellectual functions.

**Table 1:** Diagnostic criteria for Autism Spectrum Disorder

	Social communication	Restricted repetitive behavior
Criteria	Persistent deficits in social communication and social interaction across multiple contexts, currently or by history	Restricted, repetitive patterns of behavior, interests, or activities, as manifested by at least two of the following:
Illustrative examples of symptoms	(1) Deficits in social-emotional reciprocity, ranging from abnormal social approach and failure of normal back-and-forth conversation, to reduced sharing of interests, emotions, or affect, to failure to initiate or respond to social interactions (2) Deficits in nonverbal communicative behaviors used for social interaction, ranging from poorly integrated verbal and nonverbal communication, to abnormalities in eye contact and body language or deficits in understanding and use of gestures, to a total lack of facial expressions and nonverbal communication (3) Deficits in developing, maintaining, and understanding relationships, ranging from difficulties adjusting behavior to suit various social contexts, to difficulties in sharing imaginative play or in making friends, to absence of interest in peers	(1) Stereotyped or repetitive motor movements, use of objects, or speech. (2) Insistence on sameness, inflexible adherence to routines, or ritualized patterns of verbal or nonverbal behavior (3) Highly restricted, fixated interests that are abnormal in intensity or focus (4) Hyper- or hyporeactivity to sensory input or unusual interest in sensory aspects of the environment
	Symptoms must be present in the early developmental period. Symptoms may not become fully manifest until social demands exceed limited capacities, or may be masked by learned strategies in later life Symptoms cause clinically significant impairment in social, occupational, or other important areas of current functioning These disturbances are not better explained by intellectual disability or global developmental delay	
Specifiers	With or without accompanying intellectual impairment With or without accompanying language impairment Associated with a known medical or genetic condition or environmental factor Associated with another neurodevelopmental, mental, or behavioral disorder With catatonia	

The diagnostic criteria discussed above have been developed primarily with Western participants. Even though it is regarded as heavily influenced by biological factors and a developmental condition, research has recently highlighted that social and cultural factors influence diagnostic rates and the cultural acceptability of the tools used to make the diagnoses [25]. For instance, in the United States, general developmental delays or impaired language skills are common symptoms that result in a diagnosis [25]. Given that the diagnosis is based on social and contextual observations, it is not surprising that phenotypes and tools do not transfer as easily to other cultures. For example, in India, language may not be typically incorporated in the diagnostic criteria as boys acquire language skills later than girls [26]. In many Asian cultures, direct eye contact with elders is viewed as a sign of disrespect, thus the reduced eye-contact as a diagnostic feature may be seen a less atypical in these cultures [25]. There is an important need for a growing body of research addressing these cross-cultural factors in the diagnosis of ASD [25, 27]. Perhaps this research will highlight the universal features of autism that reduce the influence of contextual factors in the diagnostic criteria.

The prevalence of ASDs has, however, been increasing. In Asia, the average prevalence before 1980 was ~1.9 cases per 10,000, rising to 14.8 between 1980 and 2010 [28]. A review of epidemiological studies published between 1996 and 2001, and conducted in the United Kingdom, United States and in Scandinavia and Japan, indicated that the prevalence was likely to be within the range of 30–60 cases per 10,000 [29]. More recent estimates are as high as 1 in 68, based on 8-year old children in the United States [30]. However, a combination of the broadening of diagnostic criteria previously discussed, and the methodology employed in epidemiologic surveys, including changes in the assessment process, response rates, and differences in sample size, publication year, and geographic location, suggests that it may not be informative to estimate trends over time [31]. Increases in prevalence estimates may represent changes in the concepts, definitions, service availability, and awareness of ASDs in both the lay and professional public [32]. While a recent review of epidemiologic surveys does not support differences in prevalence across geographic regions or variability based on ethnicity or socioeconomic factors, the paucity of comprehensive datasets from low-income countries impacts the ability to detect these effects [33]. Consequently, investigations of any disproportionate impact of environmental factors on prevalence relating to specific regions are difficult to characterize and the global burden of ASD is difficult to quantify.

The etiology of ASD is commonly described as a genetic predisposition combined with an environmental impact [34]. The body of research identifying genetic deletions and duplications, inherited and *de novo*, and rare and common variants in ASD is expansive. Evidence for genetic variants in the etiology of ASD includes genes involved in intellectual disability and neuropsychiatric disorder, common pathway genes and ASD-risk genes, multigenic contributions from rare or common variations, DNA mutations, and environmental effects on gene expression and/or protein function [35]. Rare genetic risk factors, including those resulting in ASD-related syndromes (e.g. Fragile X), chromosomal abnormalities, and penetrant genes are estimated to contribute to ~20% of ASDs [35]. At least 5% of non-syndromic, idiopathic, and primarily simplex ASD are caused by *de novo* copy-number variants [36]. It is estimated that 400–1000 genes are likely to lead to a susceptibility to autism [37, 38]. Genetic influences are thought to converge on a smaller number of key pathways and developmental stages of the brain [39]. Despite the extensive research in this field, the genetic etiology for at least 70% of cases of ASD remains unknown [36]. Pre-, neo-, and post-natal environmental risk factors have also been implicated [40, 41]. For example, deficits in social interaction and language and the presence of restricted and stereotyped patterns of behavior have all been demonstrated in a mouse model of maternal infection, considered a prenatal environmental risk factor for autism [42]. Decreased levels of neurotrophic factors, which support the growth, survival, and differentiation of developing and mature neurons, have been identified as an environment risk in the neonatal period [43]. In addition, during the postnatal period, it has been proposed that a vulnerable physiology may be particularly susceptible to environmental influences [44], such as the burden of organic pollutants which has been found to be associated with the severity of autism-related symptoms [45]. It is also thought that gene-environment interactions may be involved in the etiology of ASD, although the evidence to date is derived predominantly from animal models [46].

## Symptomatology, Clinical Presentation, and Severity

The symptomatology of ASD is extensive and pervasive with a variable onset that could be considered a dimensional process [47]. While ASD is considered a lifelong condition [48], there are a range of prognoses with the recent identification of an optimal outcome whereby children previously diagnosed with an ASD were no longer considered to meet the diagnostic criteria [49]. The identification of this outcome challenges the concept that ASD phenotypes are stable and insensitive to treatment and suggests that developmental trajectories can diverge significantly [50]. The classification of ASD severity is based on the required levels of support to assist with impairments in social communication and social interaction, and restricted, repetitive patterns of behavior, interests, or activities (APA 2013) (Tables 1 and 2). However, there are concerns that conceptualizations of severity based on required levels of support could result in inconsistencies when there are mixed levels of impairment across cognitive, adaptive, and autism-related symptoms and result in site-specific applications of ASD categories [51]. Symptoms associated with ASD range from slight to profound impairment where deficits can impair all daily living functions. The severity of symptoms increases when demands in certain environments exceed the individual’s capacity to function at a required level. The spectrum of need in terms of supports and services can be vast, with the ability to function across skill areas required for daily living and across the lifespan often independent of the severity of autistic symptoms. The difficulties associated with the accurate assessment of functioning, an important factor in understanding the impact of severity on outcomes, is currently being addressed with the development of the International Classification of Functioning, Disability and Health core sets for ASD [52]. The core set is a shortlist of categories selected to encompass aspects of functioning most relevant when describing a person with ASD.

**Table 2:** Current severity specifiers for Autism Spectrum Disorder

Severity level	Social communication	Restricted, repetitive behaviors
*Level 3* Requiring very substantial support	Severe deficits in verbal and nonverbal social communication skills cause severe impairments in functioning, very limited initiation of social interactions, and minimal response to social overtures from others	Inflexibility of behavior, extreme difficulty coping with change, or other restricted/repetitive behaviors markedly interfere with functioning in all spheres. Great distress/difficulty changing focus or action
*Level 2* Requiring substantial support	Marked deficits in verbal and nonverbal social communication skills; social impairments apparent even with supports in place; limited initiation of social interactions; and reduced or abnormal responses to social overtures from others	Inflexibility of behavior, difficulty coping with change, or other restricted/repetitive behaviors appear frequently enough to be obvious to the casual observer and interfere with functioning in a variety of contexts Distress and/or difficulty changing focus or action.
*Level 1* Requiring support	Without supports in place, deficits in social communication cause noticeable impairments. Difficulty initiating social interactions, and clear examples of atypical or unsuccessful responses to social overtures of others. May appear to have decreased interest in social interactions	Inflexibility of behavior causes significant interference with functioning in one or more contexts Difficulty switching between activities. Problems of organization and planning hamper independence

Interestingly, the onset of ASD symptoms has been a focus of research that has identified an early onset pattern and a regressive onset pattern in which children appear to develop typically before losing skills and developing autism-like symptoms [53]. However, in-depth review of these conceptualizations concludes that the onset of ASD, or symptom emergence, is better considered a dimensional process and a continuum in which the early onset and regression patterns describe two extremes [47].

## Heterogeneity

Heterogeneity in etiology, phenotype, and outcome are hallmarks of ASD. These factors contribute to a clinical heterogeneity which manifest as diverse deficits or impairments in behavioral features and communicative functioning. The marked heterogeneity of ASDs has led to suggestions that rather than a single disorder, it could be constructive to reframe ASDs as ‘the autisms’, thereby giving consideration to multiple etiologies and distinct clinical entities [54]. The heterogeneity of clinical entities is in part a function of the multiple genes involved, the myriad of environmental factors impacting the developmental course of symptom expression, and the co-occurrence of medical and mental health dysfunctions in ASDs. Heterogeneity complicates the quest for personalized medicine in ASD. Three factors contributing to the heterogeneity of ASD, genetic variability, comorbidity, and gender, are now considered.

### Genetic Variation in ASD

Genetic variability is considered a major contributor to the heterogeneity of ASD. High-throughput genomic methods are rapidly increasing the pool of ASD genes and in doing so expanding the genetic variability associated with ASD heterogeneity [55]. Large datasets have not identified significant genome-wide associations with specific common variants, and associated analyses suggest that common variants exert weak effects on the risk for ASD [56]. The genetic architecture in ASD varies substantially, from a single penetrant mutation being enough to cause ASD, to an accumulation of over one thousand low-risk alleles [57]. Rare variants affecting ASD risk collectively encompass hundreds of genes [58], while copy-number variant data and *de novo* protein-altering mutations suggest extreme locus heterogeneity [59]. Furthermore, the combined effect of common low-impact genetic variants has also been associated with ASD [60]. Large numbers of genes implicated in ASD are thought to converge on common pathways affecting neuronal and synaptic homeostasis [61], and play critical roles in fundamental developmental pathways [39, 59]. For example, mutation of a single copy of SHANK3, a synaptic scaffolding protein, has been associated with language and social communication impairment in individuals with ASD [62]. In contrast, pleiotropic effects have been identified whereby the same deleterious genetic variant increases the risk for ASD and other neuropsychiatric syndromes [63, 64]. Finally, findings from pathway network analyses of gene ontologies suggest that, in addition to contributing to the core features of ASD, associated genes may contribute to vulnerabilities in important molecular mechanisms leading to multiple systemic comorbidities that also overlap with other conditions [65].

### Comorbidity in ASD

Characterizing the heterogeneity of ASD is further complicated by the occurrence of comorbidities. A recent study described comorbidities of > 14,000 participants with an ASD and highlighted the burden of comorbidity across multiple health care systems [66]. Comorbid psychopathologies significantly over-represented in ASD include anxiety [67], depression [68], ADHD [69], and intellectual disability [5, 7]; and medical comorbidities include seizures [70], sleep difficulties [71], gastrointestinal disorders [72], mitochondrial dysfunction [73], and immune system abnormalities [74].

The presence of one or more of these comorbidities is likely to be associated with more severe autism-related symptoms. For example, 11%–39% of individuals with ASD also have epilepsy and these individuals are more likely to have severe social impairments than those diagnosed with ASD only [75]. Comorbid sleep disturbance is indicated in 50%–80% of children with ASD and is correlated with daytime problem behaviors [76, 77]. Furthermore, sleep problems exacerbate the severity of core ASD symptoms [78, 79] and sleep disturbance is associated with behavioral dysregulation in children with ASD [80]. Aberrant behaviors are correlated with gastrointestinal problems in young children with ASD [81], and markers of mitochondrial dysfunction are significantly correlated with autism severity [73]. The role of immune system abnormalities in ASD is a significant focus of ongoing research. Altered immunity involving cytokines, immunoglobulins, inflammation, cellular activation, and autoimmunity have all been implicated in ASD [82]. Furthermore, altered levels of cytokines have been associated with the severity of behavioral impairments [83–85]. There is limited characterization of these associations between comorbidities in general and the severity of autism-related symptoms due to the complex nature of these relationships. For example, it has been proposed that precise characterization of the immune system’s role in the biology of autism requires an understanding of whether these relationships underlie the pathophysiology of ASD in a causative way, whether they create vulnerabilities to other causative factors such as pathogens, or whether a third factor underlies the pathology of ASD and the aberrant immune response in ASD [86]. Improved characterization of comorbidities is imperative for the development of a comprehensive understanding of ASD heterogeneity and may lead to the identification of distinct subgroups of ASD and subgroup-specific treatments [87].

### Gender

The male bias in ASD prevalence is most frequently reported as 4 males diagnosed to every 1 female [32, 88]. Intellectual functioning and sex-differential genetic and hormonal factors may modify this ratio [88]. Many theories have been proposed to explain the gender distribution, including the “extreme male brain” theory [89, 90]. The basis of this theory is that a normal male cognitive profile encompasses individuals who are better at systemizing (the drive to analyze or construct systems) than empathizing, and that autism can be considered an extreme of the normal male profile. A potential mechanism for this theory is an elevation of fetal sex steroids, which is supported by a recent study reporting that amniotic fluid steroid hormones were elevated in males who later were diagnosed with ASD [91]. However, in recent times a ‘female protective model’ has been proposed based on genetic studies. For example, a recent DNA study showed that girls display resilience to genetic insults in that they are more likely to have more extreme neurodevelopmentally related genetic mutations, including both copy-number variants and single-nucleotide variants, than males presenting with the same symptoms [92]. An alternative perspective is that females are under-identified and there may be a gender bias in the diagnostic criteria [93]. A large-scale study has found that females had greater impairments than males, presenting with more social communication and interaction symptoms, lower cognitive and language abilities, poorer adaptive function, and increased externalizing behavior and irritability, suggesting that females require more severe symptoms to be diagnosed as ASD [94]. However, females with ASD have been identified as having fewer repetitive behaviors than males [95], but equivalent impairments in social and communication skills [96].

There is an increased risk of ASD for a child with an older sibling who has been diagnosed with the condition. Previous investigations have estimated the recurrence risk to be between 3% and 18.7% [97]. Predictors of an ASD diagnosis in a younger sibling include male gender of the infant and the number of affected older siblings. In a large sample using a prospective design, the recurrence rate for multiplex families has been reported at 32.2% [97]. In a more recent and larger retrospective study, a 14-fold increase in ASD risk in younger siblings was found to be comparable across gestational age at birth and the child’s ethnicity, with the risk higher for younger boys regardless of the gender of the older sibling with ASD [98]. A higher recurrence risk has been identified in families with at least one affected female proband compared to families with only male probands, suggesting female protective mechanisms may be operating in families with high genetic recurrence risk rates [99].

## Treatment Options in ASD

Despite significant economic and societal costs, there are limited treatment options to ameliorate the symptoms associated with ASDs, including both symptoms related to diagnostic criteria and those that are considered to be a function of comorbid mental and medical conditions known to exacerbate the severity of presentation. While there are promising indications for new medical treatments for autism [100], a recent systematic review found that while many children with ASDs are treated with medical interventions, there is minimal evidence to support the benefit of most treatments [101]. There are numerous challenges for the identification of effective treatments for ASD. Systematic reviews highlight the possibility that genetic, environmental, cognitive, and social heterogeneity in the ASD phenotype produce highly variable study samples which reduce the potential effect size of an intervention [102]. Other factors contributing to the difficulties in identifying efficacious treatments include small sample sizes, the lack of significantly impaired study participants and the use of outcome measures that are not uniformly adopted or used as intended [102]. Cross-cultural differences, including what may be considered deviations from typical behavior in a particular culture but not in another culture, further complicate the quest for treatment options across the ASD population [103]. In addition, up to 30% of child ASD participants may respond to placebo treatments [104], which could contribute to reduced active intervention effect sizes.

Behavioral interventions undertaken early in life, using an intensive delivery format, are considered the current gold-standard treatment for behavioral symptoms associated with ASDs [105]. However, methodologically weak studies with few participants and short-term follow-ups are common in this field [106, 107]. Furthermore, early intensive behavioral interventions are expensive to implement and require extensive resources to execute effectively, making them inaccessible for many children with ASD and their families. Alternatively, only two pharmaceuticals are approved by the US Food and Drug Administration (FDA), risperidone and aripiprazole, for the treatment of symptoms associated with ASD. Risperidone, an adult antipsychotic, was approved in 2006 for the symptomatic treatment of irritability, including aggression, deliberate self-injury, and tantrums, in autistic children and adolescents. Risperidone, which acts by blocking the brain’s receptors for dopamine and serotonin, was found to be safe and effective for short-term treatment, with improvements observed in stereotypic behavior and hyperactivity [108]. However, significant side-effects are associated with risperidone use, including weight gain from increased appetite, drowsiness, and increased levels of the hormone prolactin, which is produced by the pituitary gland and which can have a feminizing effect on both females and males [109]. The frequency of side-effects appears to be dose-related [110], and while weight gain is common, somnolence more significantly influences the discontinuation of treatment [111]. In 2009, following evaluation of short-term efficacy and safety, the FDA also approved aripiprazole, a third-generation atypical antipsychotic, for the treatment of irritability associated with ASD in children and adolescents [112, 113]. Adverse events include sedation, fatigue, vomiting, increased appetite, somnolence, and tremor [114], with discontinuation commonly due to aggression and weight gain [112]. Aripiprazole is known as a dopamine system stabilizer and is less likely to elevate serum prolactin levels and induce extrapyramidal symptoms than risperidone [115].

The heterogeneity of ASD has implications for the assessment of treatment efficacy [116]. The design of treatment trials would benefit from the selection of treatment subgroups that maximize homogeneity in ways that improve the detection of efficacious interventions [87]. An improved understanding of the biological basis of the inherent heterogeneity in ASD is crucial in order to facilitate the identification of well-characterized subgroups. Investigation of underlying medical and psychopathological comorbidities associated with ASD such as immune system aberrations [82], mitochondrial dysfunction [73], gastrointestinal dysfunction [72], sleep disorders [117, 118], epilepsy [119], depression, and anxiety [120] may provide a means of characterizing the heterogeneity of ASD.

The treatment response in randomized controlled trials for ASDs continues to be primarily based on the observation of clinically relevant behaviors. Focusing entirely on behaviorally-defined diagnostic criteria and response to treatment risks a two-dimensional phenomenological approach to ASDs. The multi-dimensional aspects of presentation are increasingly recognized as aberrations in the biological pathways at the molecular and cellular levels, with alterations in circuitry linked to behavior [121, 122]. Furthermore, the limitations of the clinician paradigm as a standard for the diagnosis of ASD [24] support a move beyond this historical model to one increasingly guided by biological measurement [123]. Concurrently, there has been a greater focus on the importance of objective rather than subjective indicators of response, such as biomarkers, and the possibility of biological signatures contributing to the definition of ASD subgroups [123, 124]. Genomics, neuroimaging, and pathophysiological markers relating to mitochondrial function, oxidative stress, and immune function all offer potential as biomarkers to reduce the diagnostic heterogeneity and improve the prediction of treatment response [125].

The initiation of the Research Domain Criteria project by the National Institute of Mental Health also supports this paradigm-shift towards a diagnostic system founded on a deeper understanding of the biological and psychosocial bases of psychiatric disorders, and the requirement for research across multiple units of analysis including genes, neural circuits, and behavior [126]. Whilst this objective is still in the development phase it represents a shift towards precision or personalized medicine based on etiology and pathophysiology, which will hopefully ultimately parse out the issues contributing to heterogeneity. A precision or personalized medicine approach recognizes the importance of aligning treatment and support, care, and services to individual needs and outcomes. Individual outcomes drive community outcomes which drive societal outcomes.

## Conclusions

A concerning issue in ASD research, particularly for people with ASD and their carers requiring support, is the paucity of approved evidence-based treatment options available to ameliorate the core and associated symptoms of ASD. While the hallmark heterogeneity of ASDs may be a major contributing factor, it should not impede an understanding of ASD subgroups, associated markers of pathological states, and cross-cultural factors that are imperative to advancing this field of research. The diagnosis of ASDs continues to be entirely based on the observation of behaviors, or what is externally visible. However, there is now a greater recognition of complex symptomatology including medical and mental health comorbidities, due to the recent identification of relationships between comorbidities and the severity of autism-related symptoms. The identification of objective rather than subjective measures of response, such as biomarkers, and the possibility of biological signatures contributing to the definition of subgroups of ASDs will advance the quest for personalized medicine and treatment models in this highly heterogeneous population.
